# Comparative Analysis of Complete Chloroplast Genomes and Phylogenetic Relationships of 21 Sect. *Camellia* (*Camellia* L.) Plants

**DOI:** 10.3390/genes16010049

**Published:** 2025-01-03

**Authors:** Xu Xiao, Juyan Chen, Zhaohui Ran, Lang Huang, Zhi Li

**Affiliations:** 1 Key Laboratory of National Forestry and Grassland Administration on Biodiversity Conservation in Karst Mountainous Areas of Southwestern China, Guizhou Academy of Forestry, Guiyang 550005, China; xiaoxu199801@163.com (X.X.); huang_lng@163.com (L.H.); 2 College of Forestry, Guizhou University, Guiyang 550025, China; ranzhaohui1998@outlook.com

**Keywords:** sect. *Camellia*, chloroplast genomes, comparative genomics, phylogenetics

## Abstract

**Background**: Section *Camellia* is the most diverse group in the genus *Camellia* L., and this group of plants has a long history of cultivation in China as popular ornamental flowers and oil plants. Sect. *Camellia* plants present diverse morphological variations and complexity among species, resulting in uncertainty in the classification of species, which has resulted in a degree of inconvenience and confusion in the use of plant resources and research. **Methods**: Here, We sequenced and assembled the chloroplast genomes of 6 sect. *Camelli*a and performed comparative chloroplast genome analysis and phylogenetic studies combined with 15 existing sect. *Camellia* plants. **Results**: The chloroplast genome of 21 species in sect. *Camellia* species were quadripartite with length of 156,587–157,068 bp base pairs (bp), and a highly conserved and moderately differentiated chloroplast genome arrangement. The 21 sect. *Camellia* chloroplast genomes were similar to those of angiosperms, with high consistency in gene number, gene content and gene structure. After the annotation process, we identified a total of 132 genes, specifically 87 sequences coding for proteins (CDS), 37 transfer RNA (tRNA) genes, and 8 ribosomal RNA (rRNA) genes. The *ycf*1 gene in 21 species of the sect. *Camellia* was present only in the small single-copy/inverted repeat of a (SSC/IRa) region. Sequence variation was greater in the large single-copy (LSC) region than in the IR region, and the majority of the protein-coding genes presented high codon preferences. The chloroplast genomes of 21 plant species exhibit relatively conserved SC (single copy region)/IR (inverted repeat region) boundaries. We detected a total of 2975 single sequence repeats (SSRs) as well as 833 dispersed nuclear elements (INEs). Among these SSRs, A/T repeats and AT/AT repeats dominated, while among INEs, forward repeats and palindromic repeats predominated. Codon usage frequencies were largely similar, with 30 high-frequency codons detected. Comparative analysis revealed five hotspot regions (*rps*16, *psa*J, *rpl*33, *rps*8, and *rpl*16) and two gene intervals (*atp*H-*atp*I and *pet*D-*rpo*A) in the cp genome, which can be used as potential molecular markers. In addition, the phylogenetic tree constructed from the chloroplast genome revealed that these 21 species and *Camellia oleifera* aggregated into a single branch, which was further subdivided into two evolutionarily independent sub-branches. **Conclusions**: It was confirmed that sect. *Camellia* and *C. oleifera* Abel are closely related in Camellia genus. These findings will enhance our knowledge of the sect. *Camellia* of plants, deepen our understanding of their genetic characteristics and phylogenetic pathways, and provide strong support for the scientific development and rational utilization of the plant resources of the sect. *Camellia*.

## 1. Introduction

Section *Camellia* is the most diverse taxon in the genus *Camellia* L., with 57 species recorded in the flora [1,2,3], 55 species of which occur in China, and the remaining 2 species of which occur in Japan. China is the distribution center of sect. *Camellia* plants, which are rich in germplasm resources and are distributed mainly in subtropical areas south of the Yangtze River in China, with northern Yunnan and Jinsha River Basins being the most concentrated [4]. This group of plants has important ornamental and economic values, including *Camellia japonica* and *Camellia reticulata*, which are important parental resources for the breeding of new camellia varieties through hybridization, etc. At present, the vast majority of garden cultivars of *Camellia* L. originated from sect. *Camellia* plants [5,6]. However, this group of plants also exhibits diverse morphological variations because of the multiple geographic environments in which they are found, overlapping and interspersed species distributions, and extensive natural hybridization in their natural environments, especially as this group of plants is known as ornamental flowers and oil plants. With a long history of cultivation in China, variation (including hybridization and polyploidization), expansion of distribution, and escape into the wild under artificial influences have resulted in interspecific diversity and complexity of morphological variation, thus leading to great divergence in the understanding of the interspecific delimitation and evolutionary trends of this group of plants.

Many experts and scholars in the field of taxonomy have researched this group of plants, and there are 57 species in 2 subgroups of sect. *Camellia* in Chang Hongda’s classification system [2]. Min taxonomically considered the complex and numerous species in sect. *Camellia* according to the definition of the species, combining them into 12 species and 6 varieties [6]. However, there is a large divergence between the two classification systems. Deng adapted Chang Hong Da’s classification of sect. *camellia* species at the lineage and subgroup levels using the RAPD technique [7]. Yang demonstrated that mitochondrial matR gene sequence analysis is suitable for exploring the phylogenetic relationships of Theaceae [8]. In the studies of Ni Hui and Zheng Qian, the species of sect. *Camellia* were divided into two parts, with the majority of the species clustered in a highly supported branch [9,10], whereas *Camellia japonica* Linn., *Camellia trichosperma* Chang, and *Camellia chekiangoleosa* Hu clustered within a large branch of sect. *Paracamellia* and sect. *Oleifera* [11,12]. After an in-depth analysis of the chloroplast genome sequences of the genus Camellia, it was found that the species of sect. *Camellia* are mainly distributed over two different evolutionary branches [13]. The combined results clearly indicate that sect. *Camellia* is not a monophyletic taxon. Wu reported that sect. *Camellia* and sect. *Oleifera* are sister groups to each other, indicating that the two groups are closely related [14]. In summary, the uncertainty in the classification of sect. *Camellia* has caused a degree of inconvenience and confusion in both production and research.

The genome of chloroplasts (cp), a key organelle that performs photosynthesis in plants, exhibits haploid and maternally inherited properties in angiosperms [15,16]. The chloroplast genome has a conserved four-part structure of a large single-copy region (LSC), a small single-copy region (SSC), and two inverted-repeat regions (IRs), a structure confirmed by several studies [17]. In comparative genomics analysis of chloroplasts, their highly variable regions have the potential to be developed into population- or species-specific molecular markers (covering SSR molecular markers and DNA barcodes), which play a key role in phylogenetic analysis and species identification studies, and are of far-reaching significance for the identification, conservation and rational utilization of endangered species [18,19]. In addition, the chloroplast genome is an indispensable and important molecular tool for exploring the adaptive evolution of plants. The core protein-coding genes of the chloroplast genome encompass genes that regulate genetic information and the photosynthetic system, as well as a variety of other functional genes. Among them, photosynthetic system genes are specifically responsible for encoding ATP synthase, the Rubisco large subunit, NADPH dehydrogenase, and components of photosystems I and II [20,21]. Through adaptive evolutionary analysis, it has been found that those chloroplast genes that are closely related to photosynthesis tend to carry positively selected loci in plants growing in various types of extreme environments, and it is highly likely that these gene regions are key factors in the adaptation of plants to different ecological environments.

In recent years, the analysis of changes in chloroplast genome sequences has been widely used in phylogenetic and evolutionary biology studies [22,23]. Chloroplast genome phylogenetic studies can utilize existing molecular techniques to study the linkages between species by comparing changes in whole genomes, genes or genome segments, and differences in gene expression in different species, thereby identifying species, determining their phylogenetic relationships, revealing the rate of species evolution, and providing important insights into species evolution and phylogeny [24,25]. Up to now, the chloroplast genomes of 15 species in sect. *Camellia* have been reported. However, unfortunately, no studies have been conducted to comprehensively and comparatively analyze the chloroplast genomes of these sect. *Camellia* species. Therefore, in order to more accurately reveal the genetic differences among different species within the sect. *Camellia* and to assess their phylogenetic relationships on this basis, it is particularly urgent to comprehensively analyze the chloroplast genomes of more sect. *Camellia* plants.

In this study, we executed high-throughput sequencing of six species samples of the sect. *Camellia* collected in the field and integrated the information of 15 species covering the sect. *Camellia* from the NCBI database for comprehensive analysis. We identified the sequence structure and variation through comparative evaluation of the sect. *Camellia* genome; analyzed the codon preferences and hotspots of high nucleotide diversity in the cp genome; and constructed a phylogenetic tree of the cp genomes of 21 species to explore the phylogeny and relationships of plants in sect. *Camellia*. Through these studies, we aim to enrich the cp genome data on the genus Camellia, with a view to providing a theoretical basis and reference for the development and utilization of the plant resources of the sect. *Camellia* and the phylogenetic relationships among related species.

## 2. Materials and Methods

### 2.1. Plant Materials and DNA Extraction

In this study, a total of 21 samples from sect. *Camellia* plants were utilized. Among them, the chloroplast (cp) genome sequences of 15 samples were obtained from the NCBI database, while the remaining six samples were collected as live material from Guizhou Province, China. Mature, healthy leaves—free from pests and diseases—were gathered from wild populations, thoroughly washed, and then stored in a sealed container at −80 °C for preservation. The voucher specimens collected were deposited at the Herbarium of the Academy of Forestry Sciences of Guizhou Province, China. For the six locally collected samples, total DNA was extracted using the CTAB method. The quality of the extracted DNA was assessed through 1% agarose gel electrophoresis.

### 2.2. DNA Sequencing, Assembly, and Annotation

We sequenced the DNA samples using the Illumina high-throughput sequencing platform to obtain raw sequencing data. Subsequently, these raw data were cropped and filtered using the FASTQ 0.36 software [26], resulting in high-quality, contamination-free and clean data. Then, we compared and analyzed these data with the publicly available sequences of the genus *Camellia* in the NCBI database. To construct the chloroplast genome sequence, we used two software programs, SOAPdenovo v.2 and NOVOPlasty v.4.3.5 [27,28], for ab initio assembly. During the assembly process, we used *C. chekiangoleosa* (GenBank accession number: OP709388) as the reference genome. After completing the assembly, we annotated the six chloroplast genome sequences using the GeSeq online annotation tool (URL: https://chlorobox.mpimp-golm.mpg.de/geseq.html (accessed on 5 Aug 2024)) [29]. To improve the accuracy of the annotation, we manually checked the CDS (coding sequence) start and stop codons in the annotation files using Geneious 9.0.2 software [30] and made necessary adjustments. Also, we manually checked the type, length and anticodon names of the tRNA genes using tRNAscan SE v.2 [31] software and corrected them accordingly. Finally, to visualize the annotation information, we used OGDRAW v.1.3.163 software [32] to visualize the annotation results.

### 2.3. Repetitive Sequence Analysis

We used the Krait v.1.5.1 [33] software to detect simple sequence repeats (SSRs), with specific parameters configured as follows: single-nucleotide repeat units need to be 10 or more in length, dinucleotide repeat units at least 5, trinucleotide repeat units at least 4, and tetranucleotide, pentanucleotide, and hexanucleotide repeat units at least 3. To identify dispersed nuclear elements (INEs), we used the REPuter online tool (https://bibiserv.cebitec.uni-bielefeld.de/reputer, accessed on 16 August 2024) [34] and set the minimum repeat size to 30 bp, with a sequence identity requirement of 90% (Hamming distance of 3).

### 2.4. Chloroplast Genome Boundary Regions, Genome Comparison, and Identification of Differential Hotspots 

We used the CPJSdraw Online tool (http://cloud.genepioneer.com:9929/#/tool/alltool/detail/335, accessed on 16 August 2024) [35,36] to compare and visualize the boundary regions of 21 chloroplast genomes in detail. To explore in depth the interspecific variation among these genomes, we used the mVISTA online software (https://genome.lbl.gov/vista/mvista/submit.shtml (accessed on 16 August 2024)) [37,38,39]. A comparative analysis was performed, in which the annotation of *Camellia azalea* was used as a reference standard and LAGAN was chosen as a calibration program. In order to more precisely quantify the level of nucleotide polymorphisms in these 21 chloroplast genomes, we analyzed the nucleotide diversity (Pi) of the aligned sequence matrices using a sliding window approach in the DnaSP v.6.12.0332 software [40]. In this analysis, we set the window size to 800 bp and the step size to 200 bp. Based on the analysis results, the window regions with the top 5% of Pi values were identified as potential mutation hotspot regions.

### 2.5. Crypton Bias Analysis

Relative synonymous codon usage (RSCU) is a measure of the ratio between how often a codon is actually used and how often it should occur when it is theoretically used without preference [41]. When the value of RSCU is less than 1, it means that the codon is used less frequently and is considered a low-frequency codon; a value of RSCU greater than 1 indicates that the codon is used frequently and belongs to a high-frequency codon; and when the value of RSCU is equal to 1, it means that the use of the codon does not show any obvious preference. Since short sequences may lead to inaccurate calculations of the effective codon numbers in codon usage analysis [42], to reduce this error, this study set a minimum threshold for sequence length when selecting CDSs (coding sequences) for RSCU analysis; i.e., only CDSs with a length of not less than 300 bp were selected. Subsequently, we used CodonW 1.4.2 software to analyze these filtered CDSs for synonymous codon usage [43].

### 2.6. Phylogenetic Analyses

We downloaded phylogenetic relationship data from NCBI, containing 26 chloroplast genomes of the genus *Camellia* (Table S1), selected (*Apterosperma oblata*, GenBank NO: NC035641) as an outgroup, and phylogenetically analyzed 21 of these chloroplast genomes. In constructing the phylogenetic tree, we first used MAFFT 7 [44] for sequence comparison, followed by the maximum likelihood (ML) method in IQ-TREE v1.6.12 [45]. In order to select the optimal tree construction model, we identified the optimal models for ML analysis and Bayesian inference (BI) as K81u and GTR, respectively, based on the ATCc criterion using Phylosuite v.1.2.3 [46]. After determining the optimal model, we again performed ML analysis and BI using Phylosuite v.1.2.3. In the ML analysis, we performed the standard 1000 self-expansions test to assess the support for each branch [44]. In contrast, for BI, we performed at least 10 million generations of iterations, sampling every 1000 generations to compute posterior probabilities (PP) to assess the reliability of branches. Finally, we successfully constructed phylogenetic trees by visualizing the resulting phylogenetic relationship data using an online tool, iTOL v5 (https://itol.embl.de/ (accessed on 25 August 2024)) [47].

## 3. Results

### 3.1. Basic Structure of the Chloroplast Genome

**Figure 1:**
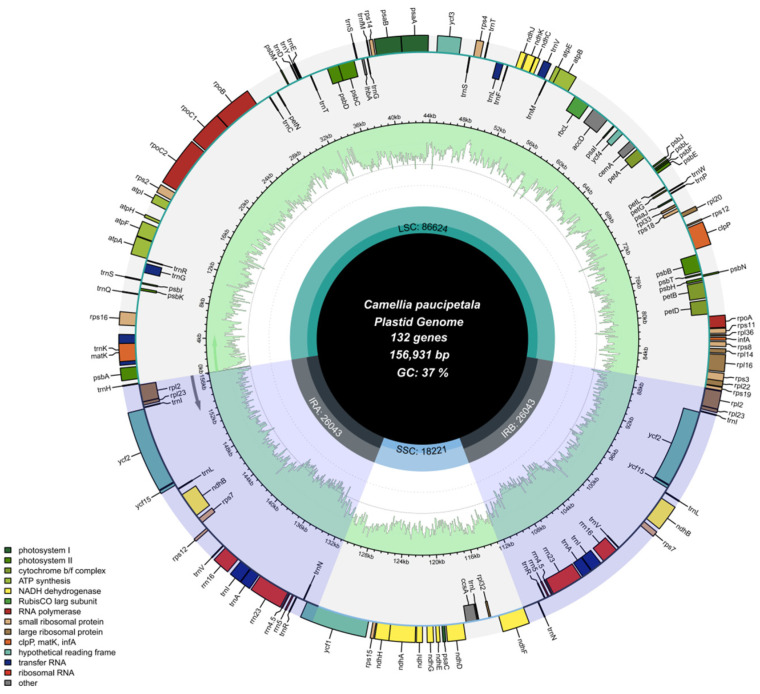
Schematic spectrum of the chloroplast genome structure (*Camellia paucipetala*).

The chloroplast genomes of all 21 species exhibited a typical tetrad ring structure, which consists of four interconnected regions (Figure 1 and Figure S1). The total sequence lengths of these genomes ranged from 156,406 to 157,039 bp, specifically comprising a large single-copy region (LSC) ranging from 86,194 bp to 86,674 bp in length, a small single-copy region (SSC) ranging from 18,194 bp to 18,415 bp in length, and a pair of inverted-repeat regions (IRs) separating the LSC from the SSC. The length of each IR region ranges from approximately 51,894 bp to 52,154 bp. The total GC content of all 21 chloroplast genomes was similar, in the range of 37.29% to 37.33% (Table S2). The GC content of the LSC and SSC regions was 35.28% to 35.34% and 30.53% to 30.62%, respectively, whereas the GC content of the IR region was significantly higher at 42.97% to 43.03%. In the protein coding sequences (CDSs), the total GC content was between 37.71% and 38.80%. Further analysis showed that the GC content at codon positions 1, 2 and 3 (denoted as GC1, GC2 and GC3, respectively) in CDSs was not uniform, showing a pattern of GC1 > GC2 > GC3, with specific values of GC1: 45.20% to 45.28%, GC2: 37.97% to 38.00%, and GC3: 29.35% to 29.44%. In addition, for these 21 chloroplast genomes, there were 132 functional genes, including 87 protein-coding genes (PCGs), 37 transfer RNAs (tRNAs) genes, and 8 ribosomal RNAs (rRNAs) genes (Table S2).

### 3.2. Repeat Sequence Analysis

**Figure 2:**
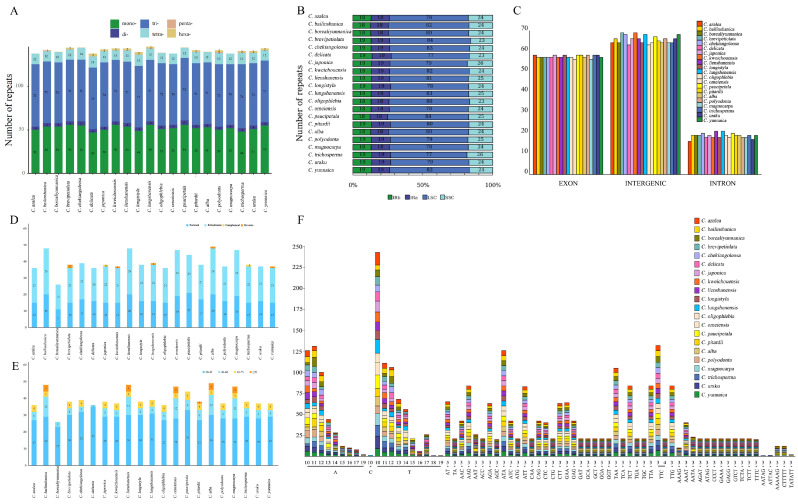
Comparative analysis of repetitive sequences in 21 chloroplast genomes of sect. *Camellia*. (**A**): the number of SSRs of six types; (**B**): the number of SSRs in the different regions; (**C**): the number of SSRs for the different gene intervals; (**D**): the number of INEs of four types; (**E**): the lengths of the INEs of the four types; from left to right, forward repeats, palindromic repeats, reverse repeats, and complementary repeats; (**F**): the number of SSRs of different repetitive units.

Simple repetitive sequences (SSRs), with their high polymorphism and stability, are considered to be ideal molecular markers in various fields such as species kinship identification, genetic diversity analysis and molecular breeding. In this study, we examined 21 chloroplast genomes for SSRs and identified a total of 2975 SSRs (Figure 2A, Table S3). Specifically, the number of SSRs per species ranged from 138 (*C. azalea*) to 146 (*Camellia lungshenensis*), and these SSRs consisted of 47 to 56 mononucleotide (Mono-) repeats, 4 to 5 dinucleotide (Di-) repeats, 70 to 74 trinucleotide (Tri-) repeats, 11 to 14 tetranucleotide (Tetra-) repeats, 0 to 1 pentanucleotide (Penta-) repeat, and 0 to 3 hexanucleotide (Hexa-) repeats (Figure 2B, Table S4). Notably, these repeat units were mainly composed of adenine (A) and thymine (T), with single-nucleotide A/T repeats being the most common, followed by dinucleotide AT/TA repeats (as shown in Figure 2F). In 21 species, we observed all six types of SSRs (Figure 2F, Table S5). In terms of regional distribution, SSRs were mainly concentrated in the LSC region, but the relative lengths of SSRs in the LSC, SSC, and IR regions did not show a clear pattern.

In addition, we analyzed the number of repetitive sequences in different gene expression types, which ranged from 138 (*C. azalea*) to 146 (*C. lungshenensis*), and which were mainly located in introns, exons, and the spacer regions of genes (Figure 2C, Table S6). Specifically, exons contained 16 to 21 repetitive sequences, introns contained 56 to 58 repetitive sequences, and the interiors of genes contained 63 to 69 repetitive sequences. On the other hand, interspersed repeat sequences (INEs) are dispersed in the genome, and they are of four types: forward repeats (F), reverse repeats (R), palindromic repeats (P), and complementary repeats (C). In this study, we detected a total of 833 INEs in 21 chloroplast genomes (Table S7), with the number of INEs per species ranging from 26 (*Camellia borealiyunnanica*) to 49 (*Camellia alba*). These INEs included 11 to 20 forward repeats, 15 to 28 palindromic repeats, 0 to 2 reverse repeats, and 0 to 1 complementary repeat. Among them, forward and palindromic repeat sequences had the highest percentage in the genome, while reverse and complementary repeat sequences were missing in some species (Figure 2D, Table S8). The length of INEs ranged from 30 to 118 bp, and it is noteworthy that a forward repeat sequence with a length of more than 90 bp was present only in *Camellia pitardii* (Figure 2E).

### 3.3. Contraction and Expansion of the IR Boundary

**Figure 3:**
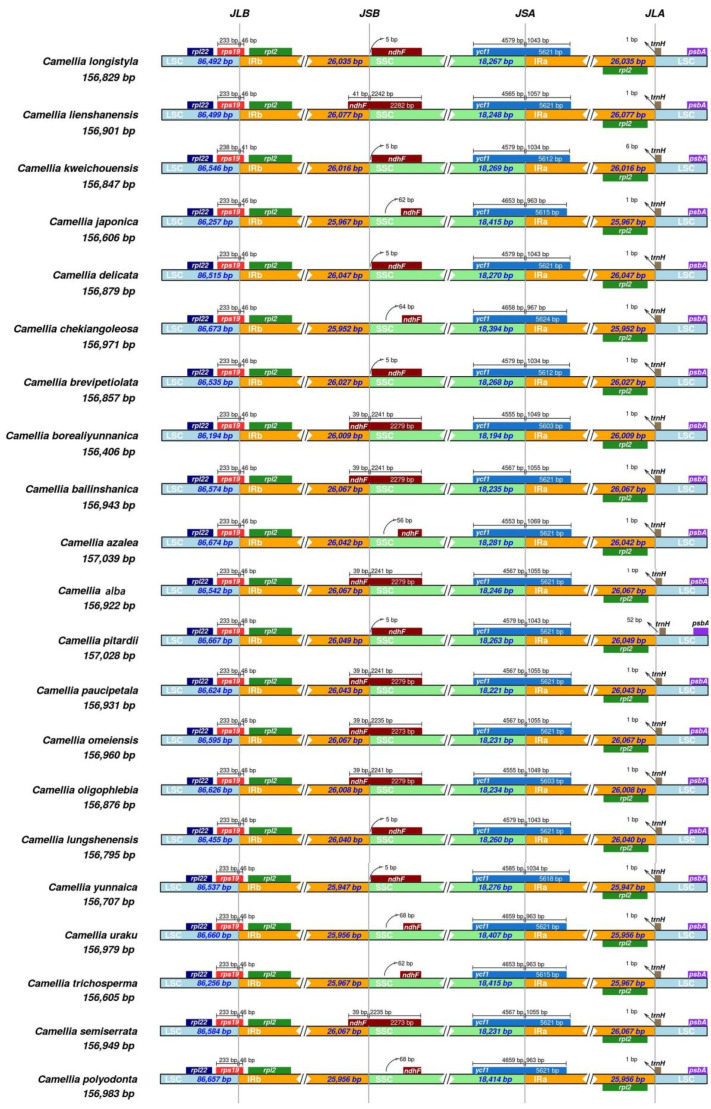
IR boundary analysis of 21 chloroplasts from sect. *Camellia*.

The 21 chloroplast genomes belonging to the sect. *Camellia* exhibit the typical structural features of a double-stranded tetrad, characterized by four distinctly defined boundaries between the SC region and the IR region. Upon comparative analysis of these boundaries, it was observed that the SC/IR boundaries across these 21 species demonstrated a high degree of conservation. Specifically, the LSC/IRB boundary, denoted as the JLB line, is precisely located within the *rps*19 gene. The IRB/SSC boundary, marked as the JSB line, is found in the overlapping segment between the ndhF gene and the pseudogene *ycf*1. Similarly, the SSC/IRA boundary, labeled the JSA line, is situated within the *ycf*1 gene, while the IRA/LSC boundary, denoted the JLA line, is positioned between the *rpl*2 gene and the *trn*H gene (Figure 3).

At the LSC/IRB boundary, the *rps*19 gene crosses the JLB line, with just a tiny fraction of its bases extending into IRB region. In the case of *Camellia kweichouensis*, the LSC portion measures 233 bp and the IRB portion is 41 bp, whereas for the other 20 species, the LSC portion remains at 233 bp but the IRB portion is 46 bp. Moving to the IRB/SSC boundary, the *ndh*F gene similarly spans the JSB line, with minimal bases venturing into the IRB region. It is worth noting that among the *ndh*F genes of eight species, *Camellia lienshanensis*, *C. borealiyunnanica*, *Camellia bailinshanica*, *C. alba*, *C. azalea*, and others, 39 to 41 bp lie to the left of the JSB line, with the remaining 2235 to 2242 bp positioned to its right.

At the SSC/IRA boundary, the *ycf*1 gene spans the JSA line, with only a negligible number of bases extending into the IRA region. Across the 21 species in sect. *Camellia*, the ycf1 gene extends between 963 and 1069 bp to the right of the JSA line, while to the left of the line, the length varies from 4453 bp to 4679 bp. Lastly, at the IRA/LSC boundary, the *rpl*2 gene sits to the left of the JLA line, while the trnH gene is positioned to its right. Specifically, the *trn*H gene of *C. pitardii* is 52 bp away from the JLA line, whereas the *trn*H genes of 20 species, including *C. lienshanensis*, *C. borealiyunnanica*, *C. bailinshanica*, *C. alba*, and *C. azalea*, are almost adjacent to the JLA line (with a distance of just 1 bp).

### 3.4. Comparative Analysis of Chloroplast Genomes

**Figure 4:**
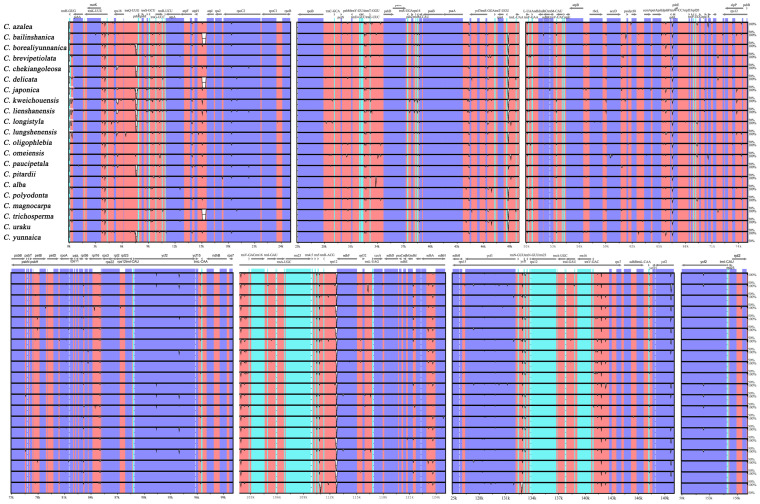
*C. azalea* was used as the reference sequence. Comparative sequence similarity analysis of the 21 chloroplast genomes.

**Figure 5:**
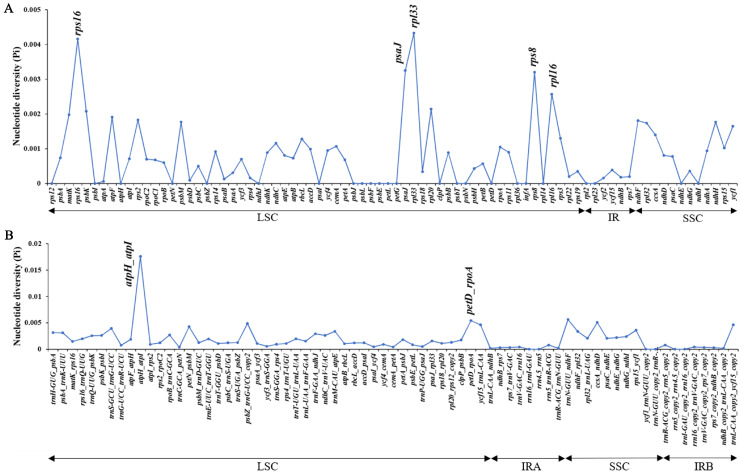
Nucleotide polymorphisms (Pi) of the 21 chloroplast genomes (window length: 800 bp; step size: 200 bp). (**A**): Nucleic acid polymorphism analysis of common genes; (**B**): Nucleic acid polymorphism analysis of common intergenic regions.

In this study, we performed multiple comparisons of the chloroplast genomes of 21 species of the red camellia group using the chloroplast genome of *C. azalea* as a benchmark, and visualized the comparison results (Figure 4). The analysis results showed that the LSC (large single copy) region exhibited higher nucleotide variability compared to the IR (inverted repeat) region, while the noncoding region also exhibited greater variability compared to the coding region. In order to more precisely quantify the nucleotide polymorphism levels of these 21 chloroplast genomes and identify the mutational hotspot regions among them, we calculated the Pi values of these genomes in the aligned matrix with a window of 800 bp, which ranged from 0 to 0.00433. Further filtering the window of the top 5% of Pi values, we identified the lowest Pi value of 0.00257 as a threshold and accordingly detected five potential molecular marker regions that were considered as mutational hotspots due to having high Pi values (>0.00257), specifically five gene regions of rps16, psaJ, rpl33, rps8, rpl16, and two gene spacer regions atpH-atpI and petD-rpoA (Figure 5, Table S9). It is noteworthy that these seven mutational hotspot regions are all located within the LSC region, where mutations such as base substitutions or deletions may lead to changes in the length of coding gene sequences, which in turn cause structural variability between coding and noncoding regions.

In addition, we also performed a Mauve comparison analysis of the chloroplast genes of 21 plant species (Figure S2) to explore whether there are phenomena such as rearrangement or insertion of fragments at gene positions. The results showed that no large segmental gene rearrangements were observed in the chloroplast genome sequences of these 21 species, indicating good covariance between them.

### 3.5. Codon Preference Analysis

Codon usage preference, which refers to the phenomenon that certain codons are used significantly more frequently than their synonymous codons in a given species, is a phenomenon that has a direct impact on the efficiency and accuracy of protein synthesis. In this study, we carefully selected at least 58 coding sequences (CDSs) in each species with a length of not less than 300 bp for in-depth analysis, and a total of 64 different codons were identified. The total number of these codons showed some variation among the 21 selected species, with the exact number ranging from 23,007 in *C. borealiyunnanica* to 23,033 in *C. lienshanensis* (Table S10). It is noteworthy that UAG (as the standard stop codon) was the least frequently used in all species, while AUU (encoding isoleucine) was the most “favored” codon, with the highest frequency of use. In terms of amino acids, tryptophan (Trp) and methionine (Met) are relatively less abundant in these species, whereas arginine (Arg), leucine (Leu), and serine (Ser) are relatively more abundant, making them the “leaders” in terms of amino acid content in these species.

**Figure 6:**
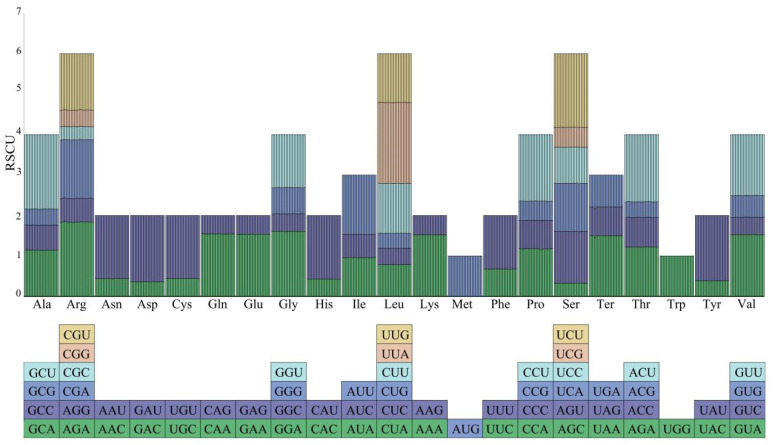
RSCU values for codons and stop codons encoding 20 amino acids in 21 chloroplast genomes. Each column in the bar graph represents one species.

To gain a deeper understanding of the usage preference of these codons, we introduced the metric of relative usage of synonymous codons (RSCU) and performed an exhaustive statistical analysis and intuitive visualization for 21 chloroplast genomes (Figure 6). The analysis results show that among the 64 total codons, 30 codons have RSCU values greater than one, which we define as high-frequency codons. It is worth noting that 29 of these 30 high-frequency codons end in A/U. On the other hand, there are 32 codons with RSCU values less than one, which we consider to be low-frequency codons, and 29 of them end with G/C. It is particularly noteworthy that two amino acids, methionine (Met, M) and tryptophan (Trp, W), are encoded only by the unique codons AUG and UGG, respectively, which both have RSCU values of one, which clearly indicates that these two amino acids do not show a specific preference in codon usage.

### 3.6. Phylogenetic Analysis

**Figure 7:**
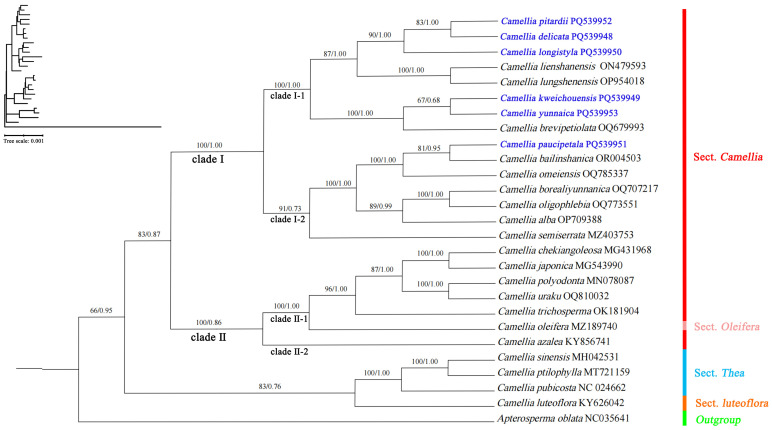
Phylogenetic tree obtained via the maximum likelihood (ML) and Bayesian inference (BI) methods for sect. *Camellia*, for 21 species on the basis of complete genomes. (Blue bold font indicates the six newly sequenced species).

In order to analyze the relationships between 21 species in sect. *Camellia*, based on the complete chloroplast genome, we constructed a phylogenetic tree using 27 cp genomes (ML and BI) (Table S1), and combined the phylogenetic trees constructed by ML and BI to illustrate the plant genetic relationships of sect. *Camellia*. (Figure 7). The phylogenetic tree with *A. oblata* as the outgroup clustered Camellia spp. into one unit, and the 21 species of sect. *Camellia* and *C. oleifera* were clustered into one unit with high support (BS = 83; PP = 0.87), suggesting that the affinity of the oil tea to plants in the red camellia group is close.

The results of the phylogenetic tree support Chang Hongda’s delineation of the two subgroups of the Hongshan Formation, with the 21 species of sect. *Camellia* divided into two clades: clade I (BS = 100; PP = 1.00) was composed of clade I-1 and clade I-2; clade I-1 (BS = 100; PP = 1) was composed of *C. pitardii*, *Camellia delicata*, *Camellia longistyla*, *C. lienshanensis*, *C. lungshenensis*, *C. kweichouensis*, *Camellia yunnaica*, and *Camellia brevipetiolata*; clade I-2 (BS = 61; PP = 0.73) was composed of *C. paucipetala*, *C. bailinshanica*, *Camellia omeiensis*, *C. borealiyunnanica*, *Camellia oligophlebia*, *C. alba*, and *Camellia semiserrata* are composed; clade II (BS = 100; PP = 0.86) was composed of clade II-1 and clade II-2. Clade II-1 (BS = 100; PP = 1) was composed of *C. chekiangoleosa*, *C. japonica*, *Camellia polyodonta*, *Camellia uraku*, *C. trichosperma*, and *Camellia oleifera*; and clade II-2 consisted of *C. azalea* alone. Among them, the most closely related clades with high support were *C. lienshanensis* and *C. lungshenensis*, which were clustered into one unit (BS = 100; PP = 1); *C. chekiangoleosa* and *C. japonica*, which were clustered into one unit (BS = 100; PP = 1); and *C. polyodonta* and *C. uraku*, which were clustered into one unit (BS = 100; PP = 1).

## 4. Discussion

After an in-depth comparative analysis of the GC content of the 21 chloroplast genomes, we observed a remarkable phenomenon: the GC content of the IR (inverted repeat) region was significantly higher than that of the LSC (large single copy) and SSC (small single copy) regions. Given the positive effect of high GC content on enhancing genome stability [13,18,48], we hypothesize that the reason that IR regions exhibit high conservation may be closely related to their GC-rich sequence characteristics. GC content is correlated with sequence stability; the higher the GC content, the more stable the sequence is, the less prone to mutation it is, and the more conserved it is. Among the four partitions of the chloroplast genome of *Camellia sinensis*, the highest average GC content is in the two IR regions; i.e., the most conserved part of the sect. *Camellia* chloroplast genome is the IR region, which is the same as that of a variety of angiosperms [49]. In order to explore the relationship between these chloroplast genomes more deeply, we conducted a meticulous comparative analysis of the chloroplast genomes of 21 species using advanced tools such as mVISTA and Mauve. The results show that these genomes exhibit good structural covariance, and we did not find any inversion or translocation of DNA fragments during the sequence comparison. This finding further confirms that chloroplast genomes are generally conserved, which is consistent with the results of existing studies [24,49].

Simple repetitive sequences are present in large numbers in living organisms and influence various life activities of organisms [50]. In this study, the chloroplast whole-genome SSR sequences of 21 species in the red camellia group were examined, and six types of nucleotide repeat units were detected, of which five-nucleotide repeats were detected in only one species, and all six types of SSRs were detected in all 21 species. The SSRs were located mainly in the LSC region. This finding supports the findings of previous studies of *C. sinensis* nucleotide repeat units in the chloroplast genome [51,52]. SSRs (Simple Sequence Repeats) are mostly in the form of single-nucleotide repeats, and in this study we found that such single-nucleotide repeats existed only in the form of A/T types and were not observed in G/C types. Single-nucleotide A/T repeats are common in most angiosperms and may be an important factor contributing to the high content of AT bases in the chloroplast genome [53,54]. The SSR loci identified in this study are important for subsequent genetic diversity analysis and phylogenetic studies of 21 plant species. Repeat sequences are rich in genetic information, and their presence is often associated with the phenomenon of mismatches in DNA strands, and there is a high degree of correlation between such mismatches and sequence variation. In the process of biological evolution, highly evolved organisms are often accompanied by the appearance of more repetitive sequences. [55,56]. Repetitive sequences contain a great deal of genetic information, and the presence of repetitive sequences can lead to DNA strand mismatches, which are highly correlated with sequence variation, and organisms with high levels of evolution tend to be accompanied by a greater number of repetitive sequences. In this study, we found that forward and palindromic repeat sequences occupied the highest proportion in the detection of long repeat sequences performed for the chloroplast genomes of 21 species, while reverse and complementary repeat sequences were missing in some species.

The chloroplast genome, with its structural simplicity, moderate evolutionary rate, and highly conserved sequences, has become an ideal tool for constructing phylogenetic relationships at multiple taxonomic levels, and numerous genes, introns, and spacer regions have been widely developed and applied for this purpose. On the other hand, DNA barcoding, as an innovative species identification technology, realizes the rapidity, accuracy and efficiency of species identification by selecting standard short gene fragments as identification markers [57]. In our study, in order to explore potential molecular markers, we performed an in-depth nucleotide polymorphism analysis of the chloroplast genomes of 21 species. The analysis showed that the genetic polymorphism of the IR (inverted repeat) region was significantly lower than that of the LSC (large single copy) and SSC (small single copy) regions in all species. In addition, we found that the noncoding portion of the coding region sequences was relatively conserved, an observation that is consistent with the findings of most angiosperms. These findings not only deepen our understanding of the genetic properties of the chloroplast genome, but also provide valuable reference information for future molecular marker development and species evolution studies [58,59,60]. However, we still identified seven highly variable regions in 21 species of sect. *Camellia*, including five regions of *rps16*, *psaJ, rpl33*, *rps8,* and *rpl16* and two gene intervals *atpH-atpI* and *petD-rpoA*, which can be used for species identification in sect. *Camellia* plants. This is the same as the results of Camellia weiningensis by Li Qian et al. [61]. The differences in the contraction and expansion of the boundaries of the IR region of different species are significant for the study of the boundary of the IR region of chloroplasts, which can provide a basis for phylogeny and species identification.

In this study, 64 codons were detected in the chloroplast genomes of 21 sect. *Camellia* species. Methionine and tryptophan have only one codon, which is the same as the previous study of sect. *Camellia* [51,54]. There were 30 high-frequency codons, and all of the high-frequency codons ended in A/U except for the codon UUG encoding leucine. In general, codon usage preferences are similar in the same species or closely related species [52,62], as evidenced by the phylogenetic relationships of the 21 plant species.

As a key research object in molecular biology, the chloroplast genome is currently leading a new trend in species lineage identification research. In particular, with leaps in sequencing technology and the significant reduction in cost, whole genome sequencing has become an indispensable tool for phylogenetic analysis. However, uneven evolutionary rates and possible horizontal shifts among genes have led to an understanding of the fact that phylogenetic trees constructed by relying only on a single or a few gene sequences often exhibit inconsistent or even conflicting topologies. This phenomenon poses significant difficulties and challenges in accurately tracing and analyzing the evolutionary history of species [63,64,65]. In this study, we used two main methods (BI and ML) to construct the phylogenetic tree. Through the combined analysis of these two methods, we arrived at findings that strongly support the treatment of tea as a separate natural taxonomic unit in the classification system of Chang Hongda and Min Tianlu. This conclusion not only deepens our understanding of the position of tea as a species on the evolutionary tree and its relationship with other related species, but also provides more solid evidentiary support for taxonomic and phylogenetic studies of sect. *Camellia* [1,6], with 21 species and *C. oleifera* clustered in a single unit with a high degree of support (BS = 83; PP = 0.87), suggesting that *C. oleifera* is closely related to sect. *Camellia* plants. This result is the same as that of Wu [14], who reported that sect. *Camellia* and sect. *Oleifera* are sister groups to each other, suggesting that the species of sect. *Camellia* are closely related to the oil tea group of sect. *Oleifera* [65]. The relationships between sect. *Camellia* and sect. *Oleifera* need to be further studied by expanding the sampling range. The results of the phylogenetic tree support Chang Hongda’s delineation of sect. *Camellia* into two subgroups (subsect. *Reticulata* and subsect. *Lucidissima*) [2], the division of the 21 species of sect. *Camellia* into two clades (clade I and clade II; BS = 83; PP = 0.87), and the results of the study by Zheng Qian and Ni Sui. *C. japonica*, *C. trichosperma,* and *C. chekiangoleosa* are clustered within a large branch of the oil tea group in sect. *Oleifera*, which is in general agreement with previous studies [10,11,66]. The fact that *C. trichosperma* and *C. semiserrata* are far apart in the phylogenetic tree does not support Min Tianlu’s proposal to merge *C. trichosperma* with *C. semiserrata* [6].

## 5. Conclusions

In this study, the chloroplast genome of 21 species of sect. *Camellia* were sequenced and compared, finding a relatively conservative tetrad structure; comparing and analyzing 21 species, all the high frequency codons ended with A/U, and five hotspot regions (*rps*16, *psa*J, *rpl*33, *rps*8, and *rpl*16) and two gene intervals (*atp*H-*atp*I and *pet*D-*rpo*A) in the gene sequence were found to be potential molecular markers; 21 species were divided into 2 typical sub-branches and 4 minor branches, and the conferring of independent classification status to some species of sect. *Camellia* was supported. In summary, these findings not only make up for the lack of data on the phylogenetic study of the chloroplast (cp) genome of sect. *Camellia*, but also provide a valuable basis and strong support for in-depth investigation of the phylogenetic relationships of sect. *Camellia*, and for solving the taxonomic controversies and identification problems.

## Data Availability

The original contributions presented in this study are included in the article/Supplementary Material. Further inquiries can be directed to the corresponding authors.

## Supplementary Materials

The following supporting information can be downloaded at https://www.mdpi.com/article/10.3390/genes16010049/s1: Table S1. NCBI serial numbers of the 27 species used for constructing the phylogenetic tree; Figure S1. Schematic spectrum of the chloroplast genome structure; Figure S2. Comparison of 21 chloroplast genome covariates. Table S2. Characteristics of the chloroplast genome of the 21 species; Table S3. The number of SSRs of six types; Table S4. The number of SSRs in the different regions; Table S5. The number of ssr for the different gene intervals; Table S6. The number of SSRs of different repetitive units; Table S7. The number of INEs of four types; Table S8. The lengths of the INEs of the four types; Table S9. Nucleotide diversity in 21 species (Pi); Table S10. RSCU values for codons and stop codons encoding 20 amino acids in 21 chloroplast genomes.
