# Supplementary figures and images for: Comparative Analysis of Complete Chloroplast Genomes and Phylogenetic Relationships of 21 Sect. Camellia (Camellia L.) Plants
Source: Genes (Basel). 2025 Jan 3;16(1):49. doi: 10.3390/genes16010049 (PMC11764880; doi:10.3390/genes16010049)

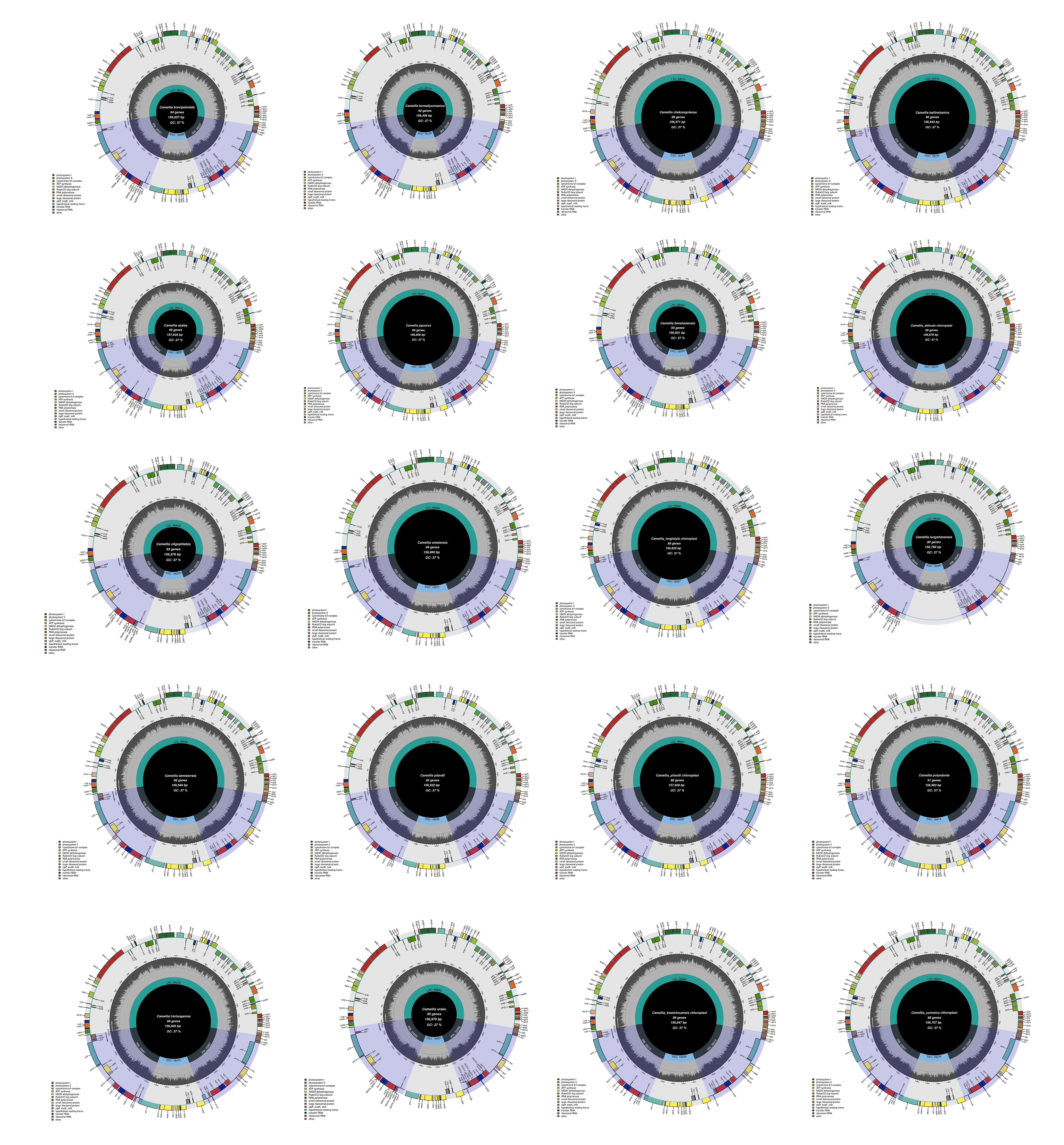

Supplement: Supplementary file 1 [file genes-16-00049-s001.zip › Figure S1-S2/Figure S1. Schematic spectrum of the chloroplast genome structure.jpg]

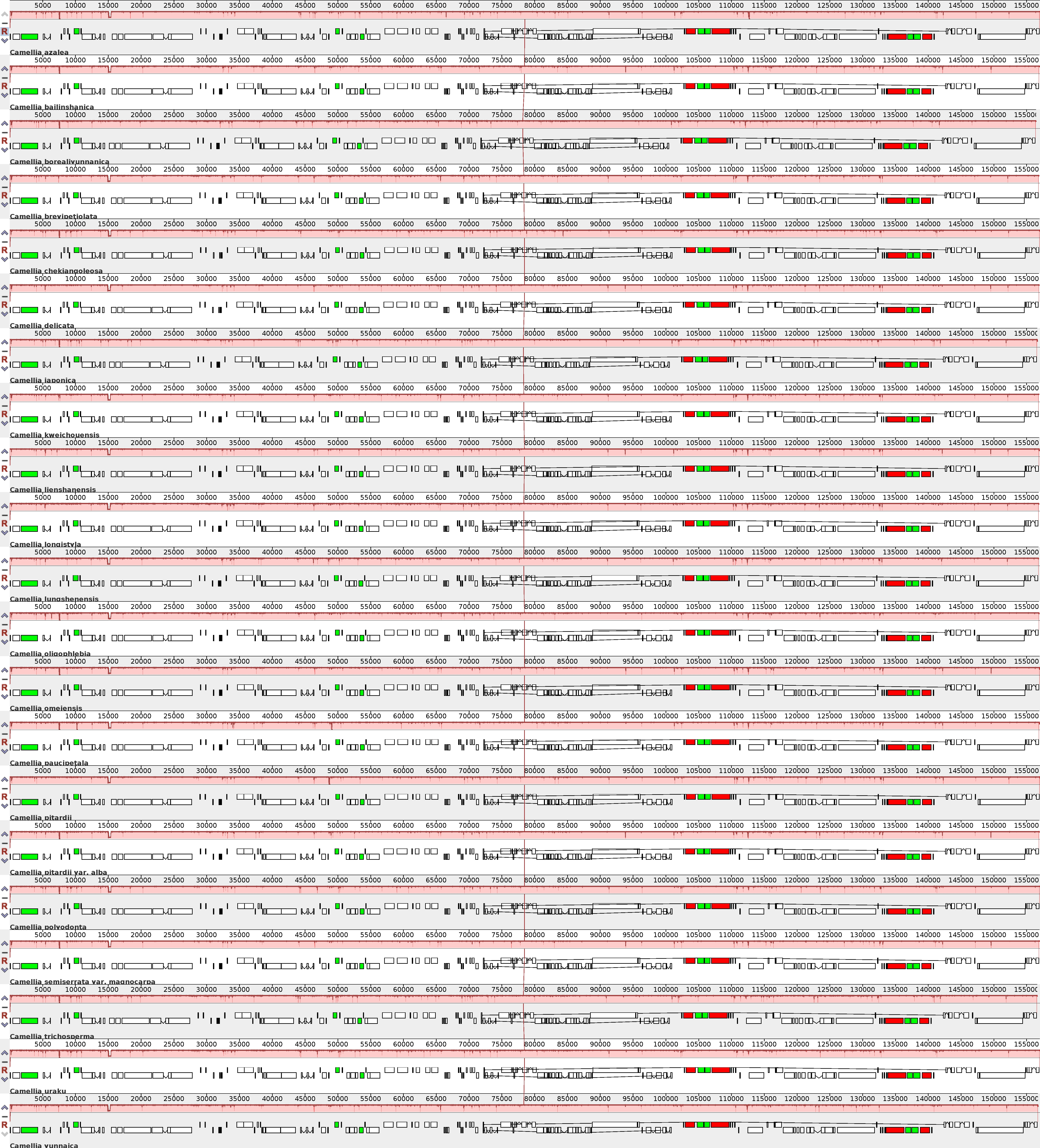

Supplement: Supplementary file 1 [file genes-16-00049-s001.zip › Figure S1-S2/Figure S2. Comparison of 21 chloroplast genome covariates..jpg]
